# Cost-effectiveness of stereotactic body radiotherapy versus conventional fractionated radiotherapy for medically inoperable, early-stage non-small cell lung cancer

**DOI:** 10.1186/s12962-023-00452-w

**Published:** 2023-07-28

**Authors:** Hui Sun, Huishan Wang, Yan Wei, Haiyin Wang, Chunlin Jin, Yingyao Chen

**Affiliations:** 1 School of Public Health, Fudan University, Shanghai, China; 2 Key Lab of Health Technology Assessment, School of Public Health, National Health Commission, Fudan University, Shanghai, China; 3 Shanghai Health Development Research Center, Shanghai Medical Information Center, Shanghai, China; 4 Department of Gastroenterology and Hepatology, Zhongshan Hospital, Fudan University, Shanghai, China; 5 Evidence-Based Medicine Center, Fudan University, Shanghai, China

**Keywords:** Non-small cell lung cancer, Cost-effectiveness analysis, Stereotactic ablative body radiotherapy, Conventional fractionated radiotherapy

## Abstract

**Background:**

Stereotactic body radiotherapy (SBRT) is a novel radio-therapeutic technique that has recently emerged as standard-of-care treatment for medically inoperable, early-stage non-small cell lung cancer (NSCLC). In this study, we compared the cost-effectiveness of SBRT with that of conventional fractionated radiotherapy (CFRT) in patients with medically inoperable, early-stage NSCLC from the perspective of the Chinese health system.

**Methods:**

A Markov model was developed to describe health states of patients after treatment with SBRT and CFRT. The recurrence risks, treatment toxicities, and utilities inputs were obtained from the literature. The costs were based on listed prices and real-world evidence. A simulation was conducted to determine the post-treatment lifetime years. For each treatment, the total costs, quality-adjusted life-years (QALYs), and incremental cost-effectiveness ratios (ICERs) per QALY were calculated. Deterministic and probabilistic sensitivity analyses were performed to assess the uncertainty of the model parameters.

**Results:**

In the base case analysis, SBRT was associated with a mean cost of USD16,933 and 2.05 QALYs, whereas CFRT was associated with a mean cost of USD17,726 and 1.61 QALYs. SBRT is a more cost-effective strategy compared with CFRT for medically inoperable, early-stage NSCLC, with USD 1802 is saved for every incremental QALY. This result was validated by DSA and PSA, in which SBRT remained the most cost-effective option.

**Conclusions:**

The findings suggested that, compared to CFRT, SBRT may be considered a more cost-effective strategy for medically inoperable, early-stage NSCLC.

**Supplementary Information:**

The online version contains supplementary material available at 10.1186/s12962-023-00452-w.

## Introduction

Lung cancer is the leading cause of cancer-related death worldwide [1]. In China, lung cancer had both the highest morbidity and mortality rates (57.26 and 45.87 per 100,000, respectively) in 2015 [2]. Non-small cell lung cancer (NSCLC) accounts for approximately 85% of all primary lung cancers [3]. Moreover, early stage I NSCLC (IA, IB) accounts for 15.3% of NSCLC cases diagnosed in China [4]. According to the Cancer Screening Program in Urban China (CanSPUC), with the implementation of low-dose chest CT screening of former and current smokers, the proportion of NSCLC cases detected in the early stage will increase [5, 6].

Currently, surgery is the standard-of-care for resectable, early-stage and functionally operable NSCLC [7, 8]. However, many patients have smoking-related cardiac or respiratory comorbidities that make them unfit for an operation. For these patients, radiotherapy represents a safer and potentially curative option [9]. Historically, patients with inoperable early-stage NSCLC were treated with conventionally fractionated radiation therapy (CFRT) at doses of 60–80 Gy in 1.8–2.0 Gy fractions. Studies have shown that patients treated with CFRT have a 5-year survival rate of 10–22% and local control rates of 50–60% [10, 11].

Based on CFRT, stereotactic body radiotherapy (SBRT) has been developed in recent years. SBRT is characterized by a high local dose of radiation given in fewer fractions as an ablative treatment, usually 10–18 Gy per treatment for 3 to 5 sessions [9]. Both prospective and retrospective studies have demonstrated that, compared with CFRT, SBRT resulted in superior local control of the primary disease without an increase in major toxicity in patients with medically inoperable stage I NSCLC [9, 12–14]. Therefore, SBRT has emerged as a new technology in the treatment of NSCLC and has been gradually incorporated into clinical practice in China [15].

Considering that the prevalence rate of NSCLC is rising and the direct medical costs per patient with NSCLC tend to increase as the disease progresses [16, 17], it is important to determine the cost-effectiveness of treatments for this disease. Nevertheless, there are few studies focused on this problem, especially in China. In this study, we used a Markov model to evaluate the cost-effectiveness of SBRT compared with CFRT for treating medically inoperable, early-stage NSCLC patients, with a view to inform clinical application and the access of medical insurance for SBRT and CFRT.

## Methods

### Decision model

A Markov model that allows hypothetical cohorts of patients to transition between different health states in a fixed increment of time was constructed in our study to simulate the clinical trajectory of patients with medically inoperable, early-stage NSCLC [18].

**Fig. 1:**
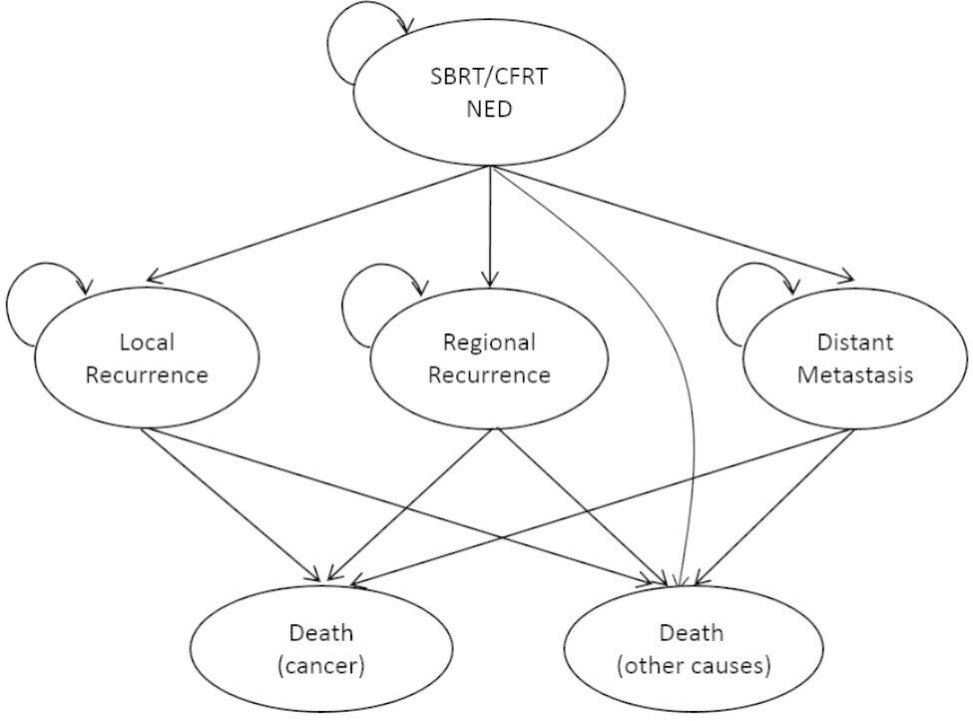
Schematic presentation of the Markov model structure for medical inoperable NSCLC treatment

We first assumed that all patients had no radiological evidence of nodal disease prior to the treatment. In the model, patients begin in the state of no evidence of disease (NED) after having receiving SBRT or conventional radiotherapy. Patients then remain in the NED state or progress to a recurrence state, including local recurrence (LR), regional recurrence (RR), and distant metastasis (DM), or death. For LR, RR, and DM, patients could remain in these states or die from the disease. Patients could die of other causes in any state in our model. The mode pattern of the Markov model is presented in Fig. 1.

On basis of the CHISEL and SPACE, two phase III randomized studies that exhaustively reported the treatment toxicity of patients who received SBRT or standard radiotherapy [9, 13], we included toxicities of grade 2 and above in the model study. For instance, patients who underwent SBRT or standard radiotherapy were mainly exposed to a risk of grade 2 pneumonitis, significant chest wall pain and grade 3 dyspnoea. For grade 2 pneumonitis, patients were treated with prednisone for 3 months. Significant chest wall pain occurred in both strategies, and it was treated with oxycodone, acetaminophen and lactulose for 6 months. If patients suffered from grade 3 dyspnoea, prednisone and home oxygen would be given for 6 months. Palliative care would be given to patients when they progressed to a recurrence state, and the treatment included doublet regimens combining cisplatin or carboplatin with vinorelbine, gemcitabine, docetaxel, paclitaxel or pemetrexed for four to six cycles. In addition, follow-up care and non-cancer end-of-life care were also included in our measurements.

The base-case modelling time horizon was set at 10 years with a cycle length of 1 year to reflect post-treatment lifetime benefit. The initial age of each patient is 74 years old, which is consistent with the SPACE and CHSEL study. Based on the World Health Organization’s recommendation, one-time and triple China gross domestic product per capital was referenced as the threshold of willingness to pay [19, 20]. The model was created and analysed with Microsoft Excel 2016.

### Model assumptions and data

#### Transition probability

The probabilities of progressing from NED to LR, RR, and DM, and the treatment related complication rates, were derived from randomized studies that compared SBRT and CFRT in medically inoperable, early-stage non-small cell lung cancer patients [9, 13]. The probabilities of transition from LR, RR, and MD to death were retrieved from a literature review [21]. The probabilities of non-cancer related death for patients with inoperable stage I NSCLC were taken from Fakiris and Andratschke et al. [22, 23]. The probability of progressing from NED to death was assumed to be similar to the general population mortality rates adjusted by age, which were obtained from the National Bureau of Statistics of China [24]. The details of the transition probability inputs in the model are summarised in Supplemental Table 1.

#### Costs

The study was conducted from the perspective of the Chinese healthcare system. Only direct costs were incorporated into the model, including the cost of radiotherapy, the cost of adverse effect management, the cost of follow-up care, the cost of palliative care, and the cost of non-cancer end-of-life care.

The total costs during radiotherapy were extracted from a real-world evidence study, which compared the cost of SBRT versus conventional radiotherapy on the basis of data from Hubei, Guangxi and Fujian provinces in China [25]. The costs of managing adverse events were determined by the market drug price across public healthcare institutions in China. The frequencies of follow-up care consisted of every 3 months for the first 2 years after radiotherapy treatment and every 6 months for the next few years. The costs of follow-up care, including outpatient visits, computerized tomography-thorax (CT) scans and full blood count tests, along with their detailed data, were taken from the Chinese medical service charge. The cost of palliative care and the non-cancer cost of end-of-life care were adapted from local published studies [26]. The costs were discounted at an annual rate of 5%. The details of costs inputs are presented in Supplemental Table 2.

#### Utilities

The utility values of the health states were taken from the related literature [27]. The estimated utility of the states of NED, recurrence (LR, RR, and DM) and death were 0.712, 0.461 and 0, respectively. In addition to the health stated utilities, the disutility due to radiotherapy-related complications was incorporated into the analysis for both the SBRT and CFRT groups. The utilities were discounted 5% annually. The details of the utilities values are available in Supplemental Table 3.

### Sensitivity analysis

One-way deterministic sensitivity analyses of the parameters were performed to evaluate the effect of adjusting the assumptions in the model. The variance of each parameter was set to their 95% confidence interval or the range reported in the literature. If both the confidence interval and reported range were unavailable, the variance was varied by 25% [28].

A probabilistic sensitivity analysis (PSA) was conducted to further explore the uncertainty of the input parameters by random sampling of the parameters from the assigned distributions. Beta and gamma distributions were selected according to the nature of the variable. Five thousand iterations of Monte Carlo simulations were applied to generate a distribution of ICER outcomes, which are shown as a scatterplot. Across a range of willingness to pay (WTP) thresholds, the cost-effectiveness acceptability curve (CEAC) was determined by calculating the probability of SBRT being cost-effective.

## Results

### Base case results

In the base-case analysis of the 10-year time horizon, treatment with SBRT was associated with a mean cost of CNY110,740 (USD16,933 (exchange 2020: $1= ¥6.54) and 2.05 QALYs, whereas treatment with CFRT was associated with a mean cost of CNY115,926 (USD17,726) and 1.61 QALYs. In comparison to the CFRT group, the SBRT group was associated with better health outcomes (with a difference of 0.44 QALYs) and lower treatment costs. Hence, SBRT is likely to be considered a more cost-effective strategy (incremental cost effective ratio [ICER]) CNY − 11,785 [USD1,802] per QALY) compared with CFRT for medically inoperable, early-stage NSCLC **(**Table 1**).**

**Table 1:** Base case results

Arm	Key results		Costs/ QALY	ICER
	Discounted costs	Discounted QALYs		
SBRT group	$16,933	2.05	8,260	Dominant
CFRT group	$17,726	1.61	11010	Comparator
Incremental	$-793	0.44		——

Abbreviations: SBRT, stereotactic body radiotherapy. CFRT, conventionally fractionated radiation therapy. ICER, incremental cost-effectiveness ratio. QALY, quality-adjusted life-years.

Among the cumulative costs of the SBRT group, the costs of non-cancer end-of-life care accounted for the largest proportion (49%) of the total costs incurred, followed by the total costs of SBRT (33%) and the costs of palliative care (10%). Similarly, the largest proportion of lifetime cumulative cost in the CFRT group was that for non-caner end-of-life care (51%), followed by the total costs of CFRT (30%) and the costs of palliative care (12%). The other costs accounted for smaller proportions.

### Deterministic sensitivity analysis

One-way DSA found the model to be robust on every assumption. The results are presented as a tornado diagram in Supplemental Fig. 1. As shown in the diagram, the factors that most strongly influenced the model outcomes were the total costs of SBRT, followed by the total costs of CFRT, while the remaining factors were ranked in decreasing order of: probability of transition from NED to local recurrence; cost of end-of-life care; probability of transitioning from LR, RR, and DM to death; discount rate; costs of palliative care for any recurrence, utility of no evidence of disease; etc. In the current study, “transitioning from LR, RR, and DM to death” ranked 6th among all influencing factors, and the model to be robust on this assumption.

### Probabilistic sensitivity analysis

**Fig. 2:**
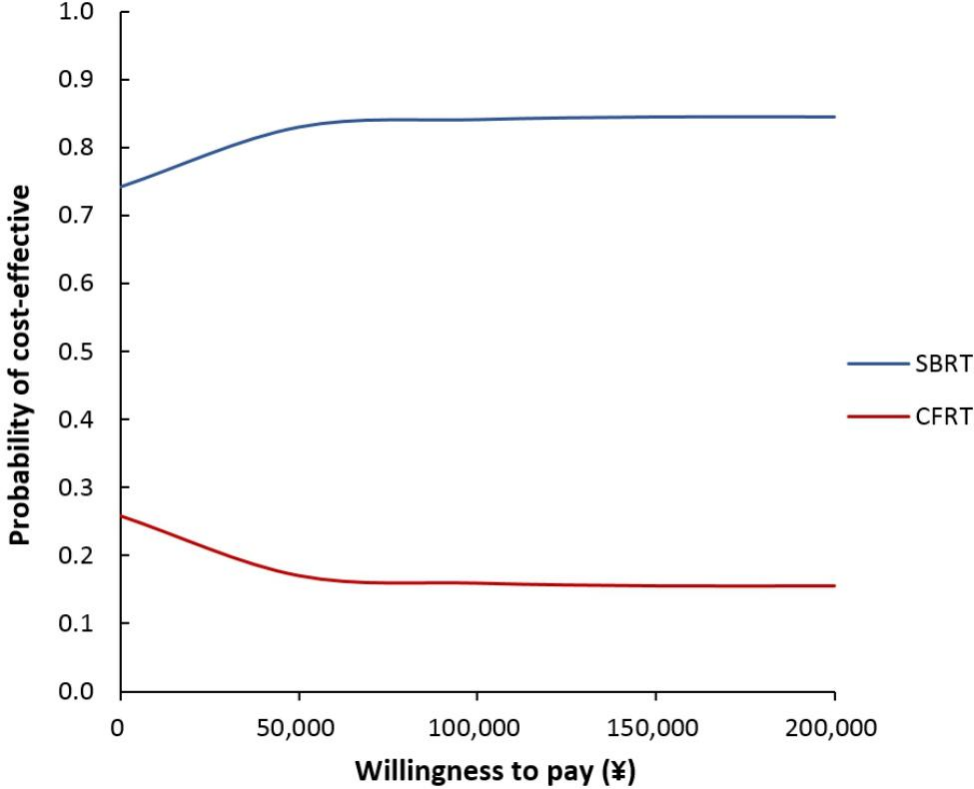
Cost-effectiveness plane showing results from 5,000 Monte Carlo simulations

**Fig. 3:**
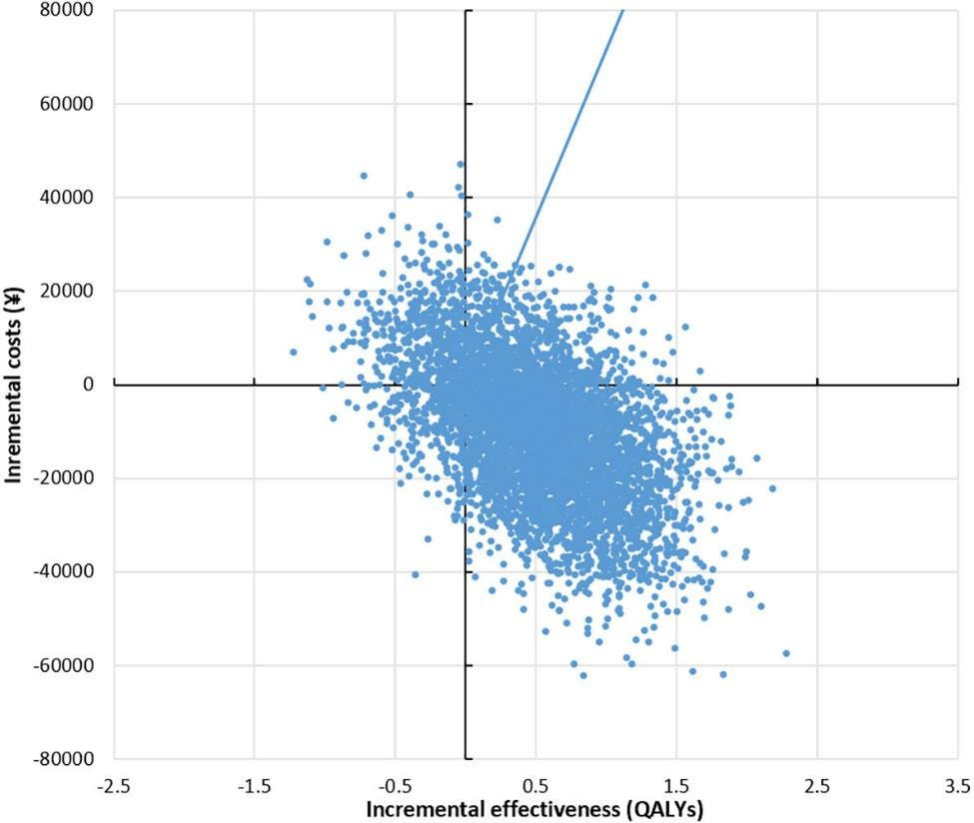
Cost-effectiveness acceptability curve of SBRT versus CFRT.

The PSA results are illustrated by the cost-effectiveness plane and the cost-effectiveness acceptability curves (Figs. 2 and 3). Each small dot represents the incremental cost and incremental QALY from one simulation. The probability that SBRT is cost-effective (calculated as the proportion of Monte Carlo simulations that fall below a given WTP threshold) is plotted against a range of WTP thresholds.

The results showed that SBRT was dominant compared to CFRT in 60.8% of 5000 Monte Carlo iterations and was cost-effective at a WTP threshold of CNY71,800 (one time GDP in 2020) per QALY gained in 78% of iterations. When the WTP threshold was increased to CNY215,400(triple the GDP in 2020) per QALY gained, SBRT was cost-effective in 80.6% of iterations. The results favoured the cost-effectiveness of SBRT in most of the scenarios.

## Discussion

To the best of our knowledge, this study is the first to evaluate the cost-effectiveness of SBRT compared with CFRT for medically inoperable, early-stage NSCLC in China. Our analyses demonstrated that the use of SBRT was associated with better health outcomes and lower treatment costs, indicating that SBRT is a more cost-effective strategy compared with CFRT in the treatment of medically inoperable, early-stage NSCLC. The results are robust over a wide range of assumptions, including the costs, efficacies, toxicities, and health utility values. One-way deterministic and probabilistic sensitivity analyses revealed that the total costs of SBRT had the largest impact on the ICERs, but SBRT remained most likely to be a more cost-effective strategy across a wide range of societal WTP levels.

In the current study, both SBRT and CFRT were calibrated for consistency with established clinical studies. The model predicted the 3- and 5-year overall survival rates were 57.3% and 28.9%, respectively, which are quite comparable to the findings of Nagata’s and Schonewolf ’s studies, in which the 3-year overall survival was 59.9% (95% CI, 49.6 − 68.8%) and the 5-year overall survival was 34.2% (95% CI, 23 − 45%), respectively [29, 30]. For patients undergoing standard radiotherapy, the model predicted 3-year LR, RR, and DM rates of 37.4%, 10.9%, and 21.4%, respectively, which are similar to the results reported by Bradley et al. of 37%, 13%, and 19%, respectively [31]. The predicted 3- and 5-year overall survival rates were 46% and 17%, respectively, which are also consistent with Wisnivesky et al. [32] and Lanni et al. [33]. Lanni et al. reported a 3-year overall survival rate of 42%, while Wisnivesky et al. reported a 5-year overall survival rate of 14%. The 3-year overall survival in our study was also consistent with Sibley et al., who reported rates of 19–55% [34]. Thus, these results suggest that the model was accurately emulating the disease process.

The findings of our study are comparable with some previous related studies in other countries. Grutters et al. [35] demonstrated that a patient treated with SBRT was associated with 2.59 QALYs and €13,871 total costs, whereas conventional radiotherapy was associated with 1.98 QALY and €22,696 total costs; SBRT was superior to conventional radiotherapy in medically inoperable, stage I NSCLC in the Netherlands. In the study of Sher et al. [36] in the US, SBRT was recognized as a more cost-effective treatment for medically inoperable NSCLC compared with three-dimensional conformal radiation therapy (3D-CRT) and radio frequency ablation (RFA); the ICER for SBRT over 3D-CRT and RFA was $6,000/QALY and $14,100/QALY, respectively. Studies conducted by Louie et al. [37] and Mitera et al. [38] in Canada revealed that SBRT was the more cost-effective treatment modality compared with conventional radiotherapy with reference to the WTP thresholds. The findings of our study are largely concordant with these results because SBRT is superior, with significantly longer progression-free survival and overall survival but without an increase in major toxicity. In contrast to the study in Grutters et al., [35]who did not incorporate the non-cancer end-of-life care in their model studies. The results in the present study revealed cost of non-cancer end-of-life care accounts for a large proportion of the total costs, which is consistent with the study of Sher et al., [36] and a possible explanation might be that most patients entering the state of death after five-year treatment.

As a new procedure, SBRT has been gradually incorporated into clinical practice for medical inoperable, early-stage NSCLC in China. The superior local control with SBRT has been validated, with often 2 times better outcomes than those achieved with conventional fractionation. However, until the present analysis, it was unclear whether SBRT is a cost-effective strategy in China. As we have shown, SBRT is a more cost-effective strategy for medically inoperable NSCLC than CFRT. Since economic evidence is increasingly recognized as an important guide for reimbursement decision making in China, these findings are important for informing resource-planning and policymaking. Moreover, the implications of this study could affect a considerable number of patients in China. First, it is estimated that 25 to 35% of early lung cancer patients are medically inoperable, and non-invasive procedures, such as SBRT, must be implemented [39]. In addition, with the introduction of CT screening, the number of patients being diagnosed at early stages will clearly increase [40].

There are some limitations that should be noted in this study. First, due to a lack of local utility data, we used the utilities reported by Doyle et al., which were originally established for advanced NSCLC patients in the United Kingdom, which may underestimate the results of the study, and thus, these utility values may not be wholly generalizable to Asian patients in China. Second, according to CHISEL study, we included toxicities of grade 2 and above which reported in the model study, which may underestimate the extent of complications that could impact the quality of life and total costs. However, from deterministic sensitivity analysis, the rate of complications for either treatment had negligible impact on the results. Third, the probability of progressing from NED to death was assumed to be similar to the general population mortality rates.

In conclusion, compared with CFRT, SBRT is almost always the more cost-effective strategy for medically inoperable, early-stage NSCLC in China. On the basis of its efficacy and costs, SBRT should be the primary treatment for this disease.

## Electronic supplementary material

Below is the link to the electronic supplementary material.

Supplementary Material 1

## Data Availability

All data generated or analyzed during this study are included in this published article and its supplementary information files.
