# Supplementary material for: Cost-effectiveness of stereotactic body radiotherapy versus conventional fractionated radiotherapy for medically inoperable, early-stage non-small cell lung cancer
Source: Cost Eff Resour Alloc. 2023 Jul 28;21:46. doi: 10.1186/s12962-023-00452-w (PMC10375662; doi:10.1186/s12962-023-00452-w)
Supplement: Supplementary file 1 — Supplementary Material 1 [file 12962_2023_452_MOESM1_ESM.docx]

**Cost-effectiveness of stereotactic body radiotherapy versus conventional fractionated radiotherapy for** **medically inoperable, early-stage non-small cell lung cancer**

***Supplementary:***

**Table1 Transition probabilities used in the model**

| **Event** | **Baseline value** | **Range for DSA** | **Distribution**  **for PSA** | **Source** |
| --- | --- | --- | --- | --- |
| **Transition probabilities, SBRT group** |  |  |  |  |
| NED to local recurrence | 10%(2y) | 4%-19% | Beta | Ball et al [9] |
| NED to regional recurrence | 7%(3y) | 5%-15% | Beta | Nyman et al [13] |
| NED to distant metastasis | 24%(3y) | 5%-50% | Beta | Nyman et al [13] |
| Chest wall pain | 38% | 0-50% | Beta | Ball et al [9] |
| Pneumonitis rate | 4% | 0-10% | Beta | Nyman et al [13] |
| Dyspnea rate | 27% | 0-50% | Beta | Nyman et al [13] |
| **Transition probabilities, CFRT group** |  |  |  |  |
| NED to local recurrence | 26%(2y) | 13%-42% | Beta | Ball et al [9] |
| NED to regional recurrence | 8%(3y) | 5%-15% | Beta | Nyman et al [13] |
| NED to distant metastasis | 23%(3y) | 5%-50% | Beta | Nyman et al [13] |
| Chest wall pain | 16% | 0-50% | Beta | Ball et al [9] |
| Pneumonitis rate | 10% | 0-15% | Beta | Nyman et al [13] |
| Dyspnea rate | 39% | 0-50% | Beta | Nyman et al [13] |
| **Probability from LR,RR,DM to death** | 70%(1y) | 50%-100% | Beta | Burdett et al [21] |
| Abbreviations: NED, no evidence of disease. LR, local recurrence. RR, regional recurrence. DM, distant metastasis. SBRT, stereotactic body radiotherapy. CFRT, conventionally fractionated radiation therapy. DSA, deterministic sensitivity analyses. PSA, probabilistic sensitivity analyses. | | | | |

**Table 2 Unit costs included in the model**

| **Costs inputs (CNY￥)** | **Baseline value** | **Range for DSA** | **Distribution**  **for PSA** | **Source** |
| --- | --- | --- | --- | --- |
| **Cost of initial treatment** |  |  |  |  |
| Total cost for SBRT | 34,740 | 26,055-43,425 | Gamma | Zi et al [25] |
| Total cost for CFRT | 33,057 | 24,793-41,321 | Gamma | Zi et al [25] |
| **Cost of managing adverse event/month** |  |  |  |  |
| Chest wall pain | 275 | 206-344 | Gamma | a |
| Pneumonitis | 560 | 420-700 | Gamma | a |
| Dyspnea rate | 850 | 640-1,060 | Gamma | a |
| **Cost of follow-up care/visit** |  |  |  |  |
| Consultation visit | 10 | 7.5-12.5 | Gamma | b |
| CT scan | 360 | 270-450 | Gamma | b |
| Full blood count test | 90 | 68-112 | Gamma | b |
| **Cost of palliative care** | 16,241 | 12,181-20,301 | Gamma | Zhang et al |
| **Cost of end-of-life care** | 11,400 | 8,550-14,250 | Gamma | Xiong et al |
| a, treatment costs were based on selling price across public healthcare institution.  b, Costs were taken from Chinese medical service charge.  Abbreviations: SBRT, stereotactic body radiotherapy. CFRT, conventionally fractionated radiation therapy.  All costs are expressed in 2021 Chinese yuan and discounted at 5% each year. | | | | |

**Table 3 Utilities used in the model**

| **Event** | **Baseline value** | **Range for DSA** | **Distribution**  **for PSA** | **Source** |
| --- | --- | --- | --- | --- |
| **Utility inputs** |  |  |  |  |
| NED | 0.712 | 0.641-0.783 | Beta | Doyle et al [27] |
| LR RR DM | 0.461 | 0.2-0.576 | Beta | Doyle et al [27] |
| Pneumonitis, Dyspnea | 0.576 | 0.461–0.712 | Beta | Doyle et al [27] |
| Chest wall pain | 0.557 | 0.461–0.712 | Beta | Doyle et al [27] |
| Abbreviations: NED, no evidence of disease. LR, local recurrence. RR, regional recurrence. DM, distant metastasis. SBRT, stereotactic body radiotherapy. CFRT, conventionally fractionated radiation therapy. DSA, deterministic sensitivity analyses. PSA, probabilistic sensitivity analyses. | | | | |


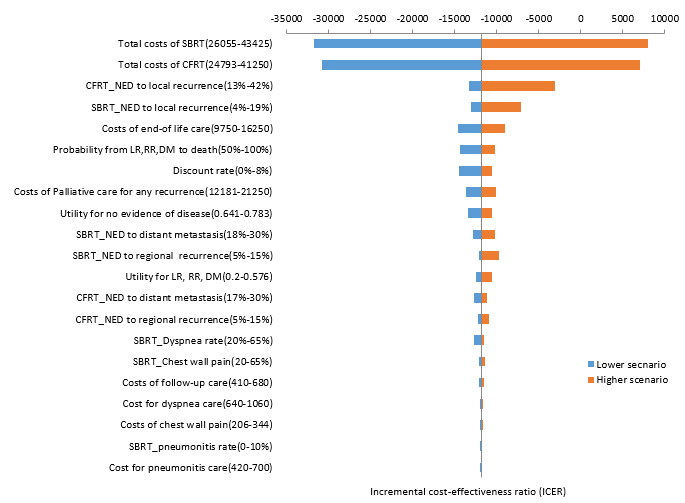


Figure1. One-way sensitivity analysis tornado diagram
